# Massive influx of victims: staff preparedness and facility readiness of Tunisian general University Hospitals.

**DOI:** 10.1016/j.afjem.2022.11.004

**Published:** 2022-12-01

**Authors:** Hamdi Lamine, Naouefel Chebili, Chekib Zedini

**Affiliations:** a Faculty of Medicine Ibn El Jazzar of Sousse, Sousse, Tunisia; b University of Sousse, Sousse, Tunisia; c SAMU03, University Hospital of Sahloul, Sousse, Tunisia

**Keywords:** Preparedness, Tunisian university hospitals, Lived experience, Massive influx of victims

## Abstract

**Introduction:**

Hospital preparedness for a massive influx of victims relies, to a certain extent, on actions, programs, and systems that are created and executed ahead of time, but also on the knowledge, skills, and professional competences of the hospital's staff.

**Aim:**

This study aims to understand the factors influencing the preparedness of Tunisian University Hospital staff in facing a massive influx of victims.

**Methods:**

This is a multi-method qualitative descriptive study conducted in nine general University Hospitals in Tunisia. The first component was a phenomenological design via open-ended interviews. The second component was a qualitative observational non-participatory design via field observations.

**Results:**

17 participants were recruited in an intentional, non-probabilistic way. Participants to this study discussed issues related to the material and financial resources of their hospitals as well as the psychological impact of managing an influx of victims. They also discussed their training, their involvement in the process, and the norm versus the circumstances in the field which led to the conclusion that: "For multiple reasons, the Tunisian University Hospitals are not ready to properly manage a massive influx of victims".

**Conclusions:**

This multi-method qualitative study discussed the factors that affected the preparedness of staff and readiness of University Hospitals included, which were mainly resources (material and financial), psychological burden, lack of training, lack of involvement in the process, and issues related to evidence-based practice. These findings support the idea that more research and more practical interventions needs to be performed to increase the preparedness level of Tunisian University Hospitals and their staff.

## African relevance

• To the authors’ knowledge, this is the first study to discuss hospital staff's preparedness in Tunisia and in the Maghreb region or north Africa.
• Understanding the level of hospital preparedness in African countries is as important as in other countries, knowing that in 2020 and in Africa alone, floods (for example) displaced seven million residents and killed 1273 people, the largest number since 2006.
• The factors affecting hospital staff preparedness in Tunisia can be extrapolated to the whole continent to draw lessons and enhance the preparedness level of African health systems.
• Being the first study in a North-African context may help initiate more research in terms of emergency preparedness and response as well as hospital safety in other regions in Africa.

## Introduction

For decades, the World Health Organization (WHO) has stressed the need for healthcare institutions to prepare their structure and personnel, including nurses and physicians, among others, to deliver the best healthcare services during health crises [1,2]. Hospital's preparedness for a massive influx of victims, disasters, or any crisis that might occur relies, to a certain extent, on actions, programs, and systems that are created and executed ahead of time, but also on the knowledge, skills, and professional competences of the hospital's staff.[3] In the last few years, the COVID-19 pandemic has shown that healthcare systems worldwide need more attention, and that more work has to be done regarding their preparedness level, specifically among the human resources that didn't feel well prepared for such a huge health crisis [4,5].

Thus, and to base intervention on scientific evidence, this study was conducted aiming to understand the factors influencing the preparedness of staff of Tunisian University Hospitals (UHs) and readiness of these facilities in facing a massive influx of victims, by exploring the lived experience of the staff members and connecting it to their context.

## Methods

### Study Design

This is a multi-method qualitative study. The first component was a phenomenological design, exploring via interviews the meaning of the lived experience of Tunisian UHs staff in managing a massive influx of victims. The second component was a qualitative observational non-participatory design via field observations of the UHs included.

### Setting

This nationwide study was conducted from November 2020 to April 2021. All UHs with a general emergency centre and a bedding capacity were included. Specialised care UHs were excluded. Among these facilities, staff members were selected purposefully based on their involvement in the emergency response activities of the hospital, to talk about their experiences in managing mass influxes of victims. Additionally, field visits to the facilities were done.

### Data Collection and Handling

Data for the phenomenological component of the study were collected using open-ended interviews conducted at the hospitals, based on an interview guide that was developed beforehand to ask specific questions about the experience of staff in a massive influx of victims. Interviews were conducted in Tunisian dialect, then meaningful quotes were translated into English for publication. Data for the observational component of the study was collected following the WHO tool 'Hospital Safety Index (HSI)'. Both data collection techniques were performed simultaneously in each hospital.

### Data Analysis

The analysis of the phenomenological part of this study was inspired from the content analysis technique developed by Giorgi[6]. This analysis was performed manually. The themes were extracted deductively, and clustered at the end. To guarantee quality of the analysis, the researcher kept an open attitude, with multiple readings and re-readings of the interviews. The logbook was used appropriately and the response with the participants was validated, and data was added, if necessary, at the end of each interview. The data derived from both collection techniques were triangulated to give more details and to contextualise the lived experience of the UH's staff.

### Ethical Considerations

After having had the authorization of the ethics committee and the Ministry of Health, all necessary information was provided to the various establishments, in a free and informed manner. The authorization of all the directors of the establishments was collected. The directors were informed of the objectives of the study and the confidentiality measures undertaken.

The verbal agreement of the participants was obtained after having discussed with them the objectives of the study, the confidentiality measures undertaken, as well as the advantages, and disadvantages of their participation. Additionally, they were informed of the possibility of withdrawing from the research project, if they so wish, at any time and without conditions.

Firm coding was assigned to each participant to respect the anonymity and confidentiality of the data. The participant's name was replaced with a code known only to the principal investigator during transcription.

## Results

### Characteristics of Participants

17 participants (six doctors, four nurses and seven technicians) were recruited, in an intentional non-probabilistic way.

The average years of experience of the participants is twelve years; the date of the first participation in the management of mass influxes of victims varies between 1993 and 2020. Table 1 illustrates the socio-demographic data of the participants (randomly coded from P1 to P17).

**Table 1:** Sociodemographic characteristics of participants.

Participants	Function	Gender	Years of experience	Date of first participation in a mass influx management
P1	Caregiver	Male	21	2000
P2	Caregiver	Female	12	2011
P3	Caregiver	Female	25	1998
P4	Caregiver	Female	8	2016
P5	Caregiver	Female	12	2011
P6	Caregiver	Female	10	2015
P7	Caregiver	Male	29	1993
P8	Caregiver	Female	2	2020
P9	Caregiver	Male	18	2005
P10	Caregiver	Male	22	1999
P11	Non-Caregiver	Male	9	2015
P12	Non-Caregiver	Female	4	2020
P13	Non-Caregiver	Female	5	2019
P14	Non-Caregiver	Male	10	2011
P15	Non-Caregiver	Female	3	2020
P16	Non-Caregiver	Male	6	2020
P17	Non-Caregiver	Male	8	2020

### The Experience of Participants

The analysis of the verbatim resulted in the emergence of 6 themes. Some themes are made up of a set of sub-themes (Table 2). An essence of the phenomenon that was revealed in this study is that: “For multiple reasons, the Tunisian University Hospitals are not ready to properly manage a massive influx of victims”.

**Table 2:** Themes identified.

Themes	Definition
Material resources	Material resources issues affecting the staff's preparedness.
Psychological impact	Psychological effects of managing a massive influx of victims.
Training	Types of training the staff had or needed to be prepared for a massive influx of victims.
Involvement	Issues related to staff's involvement in the process of management of massive influx of victims.
Financial resources	Financial resource issues affecting the staff's preparedness.
The norm versus the circumstances	The gaps between the theory and practice detected by the participants.

The themes identified are presented and explained in Table 2.

### Material Resources

#### Shortage

According to P4 “Tunisian hospitals suffer from a great lack of material resources for an adequate management of the massive influx of victims”. Moreover, “the means available are not sufficient in relation to the demand and the theoretical optimal capacity of the hospital” confirm P1, P2 and P15.

#### Not Maintained

According to P11, P12 and P16, the equipment “already available in the hospitals is not properly maintained, which considerably affects its lifespan”. In fact, “periodic maintenance is planned, well documented, but is not made according to the standards” confirms P14.

#### Misuse

Additionally, “several caregivers do not know how to use certain machines properly” confirm P8 and P15, “especially machines that are a little sophisticated and which require a little developed knowledge of new technologies” explains P15.

### Psychological Impact

#### Exhaustion

All participants confirm that they suffer from burnout frequently and that “dealing with a massive influx of victims makes the situation worse” according to P3, P7 and P10. “Caregivers must always be able to respond to emergency situations while ensuring continuous quality care for other ‘non-urgent’ patients, this is a double burden which, of course, is doubly exhausting” explains P1.

#### Fear

Apart from exhaustion “the management of mass influx of victims, being unpredictable, generates a feeling and a general 'mood' of fear”, states P3 “fear of uncertainty, fear of consequences, fear of failure…” explain P1, P2 and P9. “Nursing staff are afraid of situations that they do not control, such as covid-19, or of which they do not have enough knowledge” adds P1.

#### Solidarity vs Ignorance

Also, “faced with a situation of massive influx of victims, two scenarios appear. One is to have solidarity groups which are formed to ensure good, efficient and rapid care, the other is individuals that ignore and neglect the seriousness of the situation and continue to work as if nothing happened” explains P10.

#### Training

According to the participants, training plays an important role in dealing with mass influxes of victims, “in times of crisis there is no room for improvisation, and you need to be well-trained” explains P3.

#### Initial

According to P5, P6 and P8 “the initial training is too superficial and lacks many aspects related to disaster management” moreover the initial training of the participants is “purely theoretical without practical application or simulation” explains P2.

#### Continuous

Most participants benefited from continuous training cycles in their establishments, but none of them had a training cycle dedicated to the management of mass influxes of victims. “Despite multiple requests, the hospital administration has not planned training cycles for disaster management” states P8.

#### Specific

Several participants confirmed having taken part in workshops and/or courses in terms of managing a massive influx of victims. But according to P4, P6 and P8 “workshops and courses are not always accessible to everyone’” P6 explains that “these workshops and courses are often expensive, interfere with working hours and sometimes dedicated to a very specific audience such as specialist doctors”.

### Involvement

#### Lack of Communication

All participants expressed having felt not involved in several parts of the process of managing the massive influx of victims in their establishments, this feeling was explained by P3, P10 and P14 by the fact that “intra-hospital communication is mediocre, and information does not circulate properly”. P2 adds that “in the hospital there is no clear communication and information protocol whether in times of crisis or otherwise”.

#### The Decision-Making Process

This feeling of non-involvement was also explained by the fact staff members are “not included in the decision-making process”. According to P13 “the decision is taken in closed offices without involvement of the various hospital stakeholders”. Moreover, according to P15 “it is always the doctors, sometimes the nurses, but never the technicians involved in decision-making”.

#### Lack of Debriefing

Lack of debriefing was also stated by the participants explaining that “if we don't talk together about the problems encountered, we can never evolve and we will make the same mistakes again” (P9).

#### Financial Resources

A major problem that was discussed by all participants is the financial resources of Tunisian UHs. The problem lies, according to them, at the planning level. Indeed, “the Ministry of Health does not plan a budget line for disaster management” confirm P2, P7, P11, P14 and P17.

#### The Norm versus the Circumstances

P1 explains that the nursing staff “sometimes find themselves obliged to do non-standard practices, because the circumstances require it”, this “does not put people's lives in danger but is not considered evidence-based care” (P1). In addition, “for decision-makers emergency planning is not a priority and does not require multidisciplinary participation, it is rather the caregiver affair” (P7).

#### Complementary Field Observations

To explain the experience of participants in this study, and to understand better the state of readiness of the investigated facilities, the nine UHs were observed. Table 3 illustrates their characteristics.

**Table 3:** General information and treatment capacity of the hospitals.

University Hospitals	Construction year	Surface	Total number of staff (2019)	Total number of beds (2019)	Average bed occupancy rate (2019) in normal situations
UH01	1897	140 000 m²	2545	1065	57.84%
UH02	1942	80 000 m²	2858	704	78.46%
UH03	1910	140 000 m²	1948	880	81,9%
UH04	1985	DNA	1699	547	72.04%
UH05	1899	10 078 m²	900	180	57.2%
UH06	1912	120 000 m²	2012	995	59.09%
UH07	2002	DNA	1153	414	67%
UH08	1991	13 000 m²	1647	685	72.17%
UH09	DNA	DNA	1065	494	77%

UH, university hospital; DNA, data not available

Many UHs are made up of older and newer buildings with inherently different safety standards. Most hospital buildings showed significant signs of wear and tear, including cracks, walls damage, damaged floors, and damaged foundations. Standards are not followed in most establishments because of the improvisation and poor planning of decision-makers. Moreover, usually, no needs assessment is done properly, “we are building a ward that can accommodate 50 patients and we know very well that in 10 years 200 patients will come “(P11).

All participants confirmed that alternative means of communication (e.g., portable radios and internal telephone networks) do not exist, which explains the communication problems stated earlier. Participants P1, P3, P7, P9 and P10 explained that they mainly rely on their private mobile phones for communications. Additionally, all hospitals lacked early warning systems, although P12 confirmed that “the installations might exist, but it has never been used or tested”.

Almost all hospitals did not have an emergency response and recovery plan developed or approved under the recommendations of the Ministry of Health, according to P4, P8 and P9, “most hospitals and hospital decision-makers prefer to respond to the disaster than to prepare in advance to scenarios that may not occur” and this, according to P4 and P8, “is due to cultural roots”.

None of the UHs had an emergency budget or mechanism to obtain emergency funds, which is why participants to this study stated financial resources as impactful to their preparedness. This was explained by the fact that 'the Ministry of Health does not plan a budget line dedicated to the management of emergencies or disasters'

The feeling of fear that was expressed by the participants to this study, was because all UHs were not equipped with personal protective equipments (PPE) to be used in the event of contamination by infectious agents, chemical and/or radioactive substances.

## Discussion

The outcome of this study is that: "For multiple reasons, the Tunisian University Hospitals are not ready to properly manage a massive influx of victims" this comes in line with the literature. In fact, although there are standards to help practitioners respond to emergencies, many factors like individual qualities, training, family support, and communication may have an impact on staff's confidence [7].

First, developing hospital crisis management and evacuation plans, and staff familiarisation with them are crucial elements in preparing for massive influxes of victims. But the absence of such arrangements might cause chaos and total confusion [3,[8], [9], [10]]. The lack of planning has been mentioned on multiple occasions by the participants of this study, and according to them it is impactful to their level of preparedness at different stages. In this regard, it was demonstrated in the literature that hospital staff are more likely to show up for work when they believe that their institution has a strategy and can offer enough support and safety [11,12].

Sometimes, even though the plan exists it still lacks crucial elements. In fact, the destruction of the facility itself and its resources, along with the physical and psychological state of the staff during an incident, don't seem to be considered in hospital response plans [7,13].

Secondly, the psychological impact of dealing with massive influxes of victims was stressed. Exhaustion and fear are the most impactful feelings participants have. Several studies have shown that health staff are psychologically unprepared when asked to help in crisis circumstances since the scale of the crisis exceeds their ability for work.[14], [15], [16], [17] Additionally, a caregiver who feels psychologically unprepared and unsafe may be hesitant to take risks to save others and deal with the difficulties of emergency situations [7,18]. The team attitude towards the event, was another psychological factor stated by participants, in fact the literature discussed the importance of teamwork and effective communication in achieving an organised response. Thus, assessing teamwork behaviour can be a key part of a comprehensive hospital intervention in the event of a massive influx of victims [19,20].

Thirdly, training was stated several times by participants in our study as a factor influencing their preparedness. In fact, healthcare professionals' desire to take part in disaster and emergency response has been observed to be significantly influenced by their level of education [7,14,21]. Training is a capacity building process. It is unrealistic to expect improvement in this regard if competency-based courses are not implemented [10,[22], [23], [24]]. The literature indicated that knowledge and skills need to be continuously strengthened to improve self-efficacy [11,14,[25], [26], [27], [28], [29], [30], [31], [32]].

Lastly, the gap between scientific evidence and practice has been stated as impactful to the level of preparedness of the hospital staff. Having room for personal interpretations and experience-based interventions might affect the perception of preparedness among hospital staff. If improvising becomes the norm when dealing with the unknown, this will certainly have a negative effect [33].

Limitations: The translation of quotes from the Tunisian dialect to English had the risk of losing the meaning of some terms. Thus, a retranslation to Tunisian dialect was performed by another member of the research team, and a match of the first and last versions was performed.

In this study, it was difficult to provide a sufficiently quiet environment in the hospital. But the colleagues of the participants were notified of the interview and asked to not disturb, and no significant interruptions happened in any of the interviews.

## Conclusion

This study aiming to understand the factors influencing the preparedness of Tunisian healthcare practitioners in facing a health crisis, revealed that: “For multiple reasons, the Tunisian University Hospitals are not ready to properly manage a massive influx of victims”. Many aspects were captured during this thorough multi-method qualitative analysis. This study discussed the factors that affected the preparedness of staff and readiness of UHs included. Those were mainly lack of resources (material and financial), psychological burden, lack of training, lack of involvement in the process, and issues related to evidence-based practice.

Preparedness is the key to better crisis management, and these two are strongly interlinked and affect each other in a positive or negative way. This was discussed in the finding of this study, which supports the idea that more research and more practical interventions need to be performed to increase the preparedness level of Tunisian UHs and their staff.

## Dissemination of results

Part of the results of this research have been presented in the international public health conference in October 2020 (DOI: 10.1093/eurpub/ckaa166.597) and shared with the directors of the hospitals included in the study.

## Authors’ contribution

Authors contributed as follow to the conception or design of the work; the acquisition, analysis, or interpretation of data for the work; and drafting the work or revising it critically for important intellectual content: HL contributed 70%, NC contributed 10%, CZ contributed 20%. All authors approved the version to be published and agreed to be accountable for all aspects of the work.

## Declaration of Competing Interest

The authors declare no conflicts of interest.
